# Identifying family-child activities among children with prenatal drug exposure in a Tribal Nation: Caregiver perspectives on barriers, facilitators and positive outcomes

**DOI:** 10.1371/journal.pone.0273989

**Published:** 2022-09-09

**Authors:** Helen Russette, Joshua Brown, Annie Belcourt, Kimber McKay, Niki Graham, Erin O. Semmens

**Affiliations:** 1 Center for American Indian Health, Department of International Health, Johns Hopkins Bloomberg School of Public Health, Baltimore, MD, United States of America; 2 Department of Anthropology, University of Montana, Missoula, MT, United States of America; 3 School of Public and Community Health Sciences, University of Montana, Missoula, MT, United States of America; 4 American Indian/Alaskan Native Clinical Translational Research Program, Montana State University, Bozeman, MT, United States of America; Shinshu University School of Medicine, JAPAN

## Abstract

**Background:**

Native American newborns experience high rates of prenatal drug exposure leading to devastating outcomes within Indigenous communities. Such children are at heightened risk of maladaptive outcomes if early intervention does not occur. A need exists to identify strategies that promote resilience.

**Objectives:**

Identify barriers and facilitators that families experience in family-child engagement activities across the community, culture, outdoors, and home settings to inform a cultural-sensitive and community-relevant study aimed at quantifying positive family-child engagement activities as a resilience factor in this population.

**Methods:**

Biological parents and caregivers to children, ages 0–3 years old with or without prenatal drug exposure (N = 15) were recruited from the Confederated Salish and Kootenai Tribes to participate in an in-person semi-structured interview. Data analysis consisted of research yarning and directed content analysis to collect unique stories and to identify common activities, barriers, supports and positive outcomes to families, respectively.

**Results:**

Attending multiple powwows/celebrations, swimming, and reading were the most mentioned activities. Cost and transportation were common barriers. The most common support mechanism provided was having family or friends present to participate in activities. Cultural knowledge and bonding were common positive outcomes for a child engaging in activities. A collection of stories identified both familial barriers to traditional ways of knowing and participation in community, and community-implemented efforts to bridge that gap among families with a history of drug and alcohol use.

**Conclusions:**

This study identifies potential resilience factors specific to families to children with prenatal drug exposure that reside in Indigenous communities.

## Introduction

Opioid and poly-drug use among pregnant women is an increasing epidemic [1–3]. Native American pregnant women are nearly six times more likely to have opioid use disorder compared to their non-Hispanic black counterparts [4]. The public health implications include pregnancy complications requiring prolonged hospital stays, physical risk to both moms and babies, and ultimately risk of death to both. Prenatal methamphetamine and opioid exposure are associated with neurobehavioral deficits in long-term learning and memory and externalizing (e.g., aggression, hyperactivity, disruptive) and internalizing (e.g., anxious, withdrawn, depressed) behaviors [5–7]. These can result in complex health care needs for the child and associated economic burdens placed on families and communities [8–10]. These long-term behavioral consequences among children born with prenatal drug exposure can be partly explained by the “fetal origins hypothesis”, which is a programming theory that posits having non-matching prenatal and postnatal environments leads to negative health consequences [11]. Lin et al. adapted this programming theory to children with prenatal exposure to methamphetamine or cocaine and risk of externalizing and internalizing problem behaviors. The mismatch of the highly stimulating prenatal environment (i.e., *in utero* drug exposure] with the low stimulating postnatal environment (i.e., absence of drug, parental neglect) was confirmed in that there was a significant positive association between prenatal drug exposure and internalizing and externalizing problem behaviors [12]. Early intervention for children with prenatal substance exposure with the goal of mitigating long-term consequences is warranted [13]. Although these statistics and outcomes are important public health concerns warranting attention, it is important also to share that Indigenous communities are a place of resilience and resistance to historical and contemporary traumas. Resilience provides a buffer against stressful life events by preventing or attenuating psychological distress [14]. We assert resistance occurs when stressful events do not happen at all and we can avoid the need to always be resilient. For example, children born to mothers with a history of alcohol, marijuana, or illicit substances demonstrated greater improvements in emotional and behavioral outcomes after receiving a culturally congruent home-visiting intervention compared to their counterparts that had mothers without a substance use history [15]. These study findings inform the current study in that we also will measure similar interventions that show promising child outcomes particularly among Native American children with a history of trauma residing in an Indigenous community.

The study goal was to identify barriers and supports to family-child engagement activities and positive outcomes in children residing in one Tribal Nation within northwestern Montana with established community-wide supports to promote family and community connectedness. Our two study questions are as follows: 1) What are common family-child engagement activities that families with young children with prenatal drug exposure participate in across four domains (cultural, community, outdoors, home)? 2) What are common outcomes, barriers, and supports to these activities that impact participation among this population?

## Materials and methods

The research team applied several approaches to engaging in research with the Tribal Nation that consisted of strengths-based, community-driven, and community-based participatory research (CBPR) approaches to inform and receive approval of the study design. The research team partnered with Early Childhood Services (ECS), which houses Early HeadStart, HeadStart, and home-visiting programs within the Tribal Nation. In addition, we developed a Community Advisory Team (CAT) composed of tribally enrolled and non-enrolled community members that reside within the Tribal Nation possessing expertise on the various study components. CAT members provided guidance, review, and approval of the overall study. The Salish Kootenai Institutional Review Board provided review and approval of our study, and all participants provided informed consent. In addition, the Culture Committees and Tribal Council within the Tribal Nation approved our study.

### Sampling and recruitment

We conducted an *a priori* sampling approach using a purposive sampling method [16]. The purposive sampling approach applies predetermined participant eligibility criteria, and has demonstrated that saturation of in-depth qualitative interviews is supported by the collection of 12 interviews [17]. ECS Family Advocates supported a research staff person (HR) in recruiting primary caregivers to children, ages 0–3 years, with and without prenatal drug exposure. Recruitment communications consisted of disseminating a flyer, describing the study and eligibility criteria, on several platforms (e.g., word-of-mouth by Family Advocates, email listserv to tribal employees, ECS social media, ECS office). Ms. Russette was the interviewer for this study. She is an Indigenous woman and a citizen to the Chippewa-Cree Tribe. Family Advocates have existing relationships with the study population through the ECS Home-Visiting program. Overall, Family Advocates supported recruitment and participants by offering transportation to and from interview site, offering childcare at the interview site, and being present as an emotional support. With their support, we recruited and conducted interviews within a three-week period. Participants also received $50 cash reimbursement for their time.

Participants were eligible for this study if they were at least 18 years of age, had primary custody of their child that was between ages 0–3 years, and reported having no drugs in the home to mitigate interviewer risk if an interview occurred in the home.

### Procedure and study population

Our study was conducted from January 2019 to July 2019 in partnership with ECS and guidance from the established CAT. All ECS HeadStart sites across the Tribal Nation provided space for one research staff person (HR) to conduct in-person interviews and support participation among individuals residing in rural or isolated settings. Participants contacted one research staff person (HR) by phone call or text for recruitment eligibility. Participants provided their names, contact information, child’s age, and location. All participants consented to audio-recording during their interview and being contacted for future studies. Family Advocates and research staff received training in administering informed consent, offering further verbal descriptions, and soliciting participants’ requests to clarify interview questions. The informed consent process incorporated oral and written communication of the study to participants and included contact information for both research investigators should the participants have any questions.

Each participant was assigned a unique participant ID prior to their interview. All identifying information was separated from the interviews and stored in a secure password-protected, HIPAA-compliant web-based application. Post-interview, a research staff person (HR) transcribed all interviews. NVivo 12 software was used to store data and conduct analysis [18].

### Study instrument

Study authors, CAT members, and community partners approved and contributed to the finalization of study domains, categories and items for the data collection instrument. The 34-item semi-structured interview tool consisted of open-ended questions organized by domains. These domains consisted of cultural, community, outdoors, and home settings where the child would engage in activities with their caregiver or another family member. Categories within each domain consisted of available activities, barriers that prevent activities, and facilitators to activities. Select items within categories are activities that an individual child participates in, favorite activities, specific barriers and facilitators to participate in activities for caregivers, parents and children, and positive outcomes for children engaging in activities.

Demographic and family capital information was collected and consisted of participant and child age, race, child prenatal drug exposure status, participant relationship to child, number of family members spending time with child, number of children in the home, and family type. The research instrument delineated family type into the following four categories: 1) biological parent to a child with prenatal drug exposure; 2) biological parent to a child without prenatal drug exposure; 3) Caregiver to a child with prenatal drug exposure; 4) Caregiver to a child without prenatal drug exposure.

Study objectives for the information collected from the semi-structured interviews were designed to inform: 1) the Early Caregiver-Child Engagement survey tool in the subsequent quantitative study on activities that are relevant to the community and culturally appropriate; and 2) Early Childhood Services of potential unmet needs and other information for the population they serve.

### Data analyses

A qualitative directed content analysis approach was applied to identify most mentioned activities, positive outcomes for child participating in activities, barriers to activities, and facilitators to activities [19, 20].

Research yarning is an Indigenous research methodology that occurs as an informal, relational conversation occurring within an Indigenous context [21, 22]. We applied research yarning to highlight a collection of stories that families with a history of alcohol and drug use experience in relation to their tribal identity and practice of traditional activities. Kovach (2010) describes research yarning as a conversational method similar to narrative inquiry but having the following distinctive characteristics: Linked to tribal knowledge within an Indigenous paradigm, relational, purposeful often involving a decolonizing aim, informal and flexible, protocol driven by tribal knowledge or place, collaborative and dialogic, and reflexive (P. 43). For our purposes, informal conversation occurred that was dialogic and relational to hold space for participants to tell their story [22].

The semi-structured data collection technique chosen for this study derives individual-level experiences and opinions rather than cultural-level information [23], in which the interviewer (HR) can deviate from the questions to probe for more information when participants mentioned familial barriers to participating in their traditions and community. Analysis of data occurred by family type to improve specificity of considerable potential differences between the groups due to the inherent stigma that drug-abusing mothers and fathers experience when mother is pregnant [24].

One research staff person (HR) constructed the coding scheme and reviewed it with an additional research team member (JB) that resides within the Tribal Nation. His positionality supported rich contextual interpretation of the coding scheme by confirming Indigenous ways of knowing [25]. Inter-rater reliability assessment came from comparisons by two raters (HR, JB) of classified responses to derive the overall agreement between coders using Cohen’s kappa [26]. After initial coding was completed, the second coder (JB) coded thirty percent of all interviews. Next, queries within NVivo 12 produced kappa and agreement values [18]. The overall agreement between coders was 87.8% (11.8/15), with Cohen’s kappa being significantly higher than expected by chance and representing excellent agreement.

### Dissemination plan

Dissemination efforts thus far have included presentation of study findings or sharing a summarized digital story of study findings to ECS, culture/elder committees, families participating in ECS programs, and the Tribal Council. Results were organized and summarized and synchronous audio and closed-captioning were overlaid to improve accessibility for elders and people with disabilities to fulfill our relational obligation of sharing the findings back to the community in an inclusive and tailored manner [27].

## Results

Fifteen primary caregivers were recruited and all completed in-person interviews that ranged from 45 minutes to two hours in length at various HeadStart site locations within the Tribal Nation. Demographic characteristics for all participants are provided in Table 1.

**Table 1:** Demographic and family characteristics of participants in one Tribal Nation, 2019 (*n* = 15).

Demographics	Freq. (%)
Age, participants:	
20–24 years old	1 (6.7)
25–30 years old	10 (66.7)
31–40 years old	3 (20.0)
41 and over	1 (6.7)
Race, participants:	
Native American	14 (93.3)
Caucasian	1 (6.7)
Age, child:	
< 6 months old	3 (20)
6–12 months old	1 (6.7)
13 months– 2 years old	7 (46.7)
3 years old	4 (26.7)
Race, child:	
Native American	15 (100)
**Family characteristics**	
Family type:	
Biological parents to a child with prenatal drug exposure	5 (33.3)
Biological parents to a child with no prenatal drug exposure	8 (53.3)
Caregiver to a child with prenatal drug exposure	1 (6.7)
Caregiver to a child with no prenatal drug exposure	1 (6.7)
Family members spending time with caregivers’ children in a typical week:	
1–5	4 (26.7)
6–10	7 (46.7)
11–20	3 (20)
over 21	1 (6.7)
Children (cousins, siblings) residing in the primary home of the child:	
0	3 (20)
1–2	5 (33.3)
3–5	5 (33.3)
6 or more	2 (13.3)

### Factors related to family-child activities

Participants shared several activities that they, family, and/or friends do with their child across several settings. Results are summarized with representative quotes and organized by domains in the subsequent section.

#### Cultural and community

*Activities*. Participants mentioned attending powwows/celebrations with their child, which are often annual events consisting of traditional dance and drumming and that their child participated in their local home-visiting program. Biological parents to a child with prenatal drug exposure also mentioned participating in and attending cultural crafts and games, traditional events in sacred places, annual cultural camps, and sports-related events. Biological parents to a child with no drug exposure also mentioned participating in their local Native language immersion program at ECS, and other community-sponsored events. The caregiver to a child with prenatal drug exposure also mentioned attending ECS-sponsored events.

*They do a trip to Kootenai Falls and that’s for also cultural [purposes]…language [camp], he’s a really good talker, he’s fluent. [What’s he fluent in?]: Kootenai. My mom always teaches him words*.

- Biological parent to child with prenatal drug exposure.

*Positive outcomes*. Physical activity, connectedness, cultural knowledge, and enjoying the activity were shared as positive outcomes to participating in cultural and community events.

*She really loved the powwow, so we brought her to the other ones and when we put her in the car seat to get ready to go home she would cry… I feel like it kind of soothed her. She liked the sounds, like the drumming. And the stick game, also. Also, they get to see family members*.

*[Kootenai Falls Ceremony]: He gets to learn how sacred the Kootenai Falls is to the people and the songs and the water…He’ll be like, “Yea, I get to know where our sacred lands are, and ancestors.”*

*[Cultural language camp]: He is speaking Kootenai. They’ll teach him how to do canoes out of bark. They make drumsticks. They did do “How to make drum.”*

*[Elders Week]: He gets to learn about his elders and how to treat them. Learn about how important they are. How to respect them*.

- Biological parents to child with prenatal drug exposure.

*Barriers*. Limited or no communication of events, cost, and lack of transportation were mentioned as common barriers to participating in cultural and community events. Biological parents to a child without prenatal drug exposure also mentioned family not having a cultural background, time, and conflicting schedule for events.

*I find out about it right as it’s happening so it’s kind of like “Well, I can’t go now because it’s already happening.”*

- Biological parents to child with prenatal drug exposure

*You have to have the money to just put in an order. Who has $500 dollars to put in an order? It’s for the outfit, not even including the beadwork. Which, I can bead*.

-Caregiver to child without prenatal drug exposure

*Facilitators*. Transportation, childcare or kid-friendly events, time of event, location of event, no-cost event, feeling accepted at event, family or friend presence, and community resources were common supports to participating in community and cultural events.

*I feel accepted at the People’s Center [cultural community center] when they have classes to teach you how to bead*.

- Biological parent to child with prenatal drug exposure

*For the HeadStart events, between the Family Advocates and whoever else they have, they kind of you know if families need transportation to and from activities, they try to do as much as they can to get them there to and from*.

*I like family around or at least a friend. If I didn’t, I would just stay back and not really talk to anybody*.

- Biological parents to child without prenatal drug exposure

*We had a parent this year that made her a dress…for powwow*.

-Caregiver to child with prenatal drug exposure

#### Outdoor activities

*Activities*. Participants mentioned mostly walking or running, swimming, going to the park, and berry-picking as common favorite outdoor activities they or others do with their child.

*Hiking. We go over to Trout Creek a lot*.

-Caregiver to child with prenatal drug exposure

*Walking, hiking. Being able to observe nature. I think being outside, in general, just calms her down*.

- Biological parent to child without prenatal drug exposure

*Positive outcomes*. Gaining knowledge, bonding, happy child, soothing, physical activity, and exploring energetic were common outcomes from participation in outdoor activities.

*He gets more energetic in the water. He gets excited when he sees any kind of water*.

- Biological parent to child without prenatal drug exposure

*He learns how to play with other kids at the park*.

*He’ll try to head out all quick to the park and once he gets there, he’ll be a happy boy*.

- Biological parents to child with prenatal drug exposure

*Barriers*. Safety concerns, location, community resources, cost, negative attitudes, and time as common barriers to outdoor activities.

*Swimming, if it’s in a closed environment like the pool here, it cost a lot of money to go, so I don’t feel it’s something that we can do*.

- Biological parent to child with prenatal drug exposure

*They caught one [a mountain lion] right across the highway in the park and that’s right in town*.

- Biological parent to child without prenatal drug exposure

*I don’t think people understand probably what she’s been through and why she is the way she is. She will literally go up to any stranger and probably get in the car and go home with them*.

-Caregiver to child with prenatal drug exposure

*Facilitators*. Family or friend presence, transportation, safety of activity, and community resources were common supports to doing outdoor activities.

*There’s the Buddha Gardens…they can walk around there because they’re safe*.

- Biological parent to child without prenatal drug exposure

*They like to spend time at grandma’s house…she has a closed-in backyard. There’s a playground closer to her house than there is to mine*.

- Biological parent to child with prenatal drug exposure

*It’s always nice to bring friends for the kids, like cousins*.

-Caregiver to child without prenatal drug exposure

#### Home activities

*Activities*. Participants mentioned mostly reading, having meals together, singing, cuddling, and play time as common favorite home activities they or other family do with their children.

*We’ll sing songs to him, but there’s the songs from our Jump Dances and sweat houses. Like him learning those songs*.

*Reading. I want to do that as much as I can and if other family members would do that [reading] with him that would be awesome*.

- Biological parent to child without prenatal drug exposure

*My mom will sing to her like Native songs and stuff*.

*Dinner also with family members. Like, with all of my family, like cousins, other aunts*.

- Biological parents to child with prenatal drug exposure

*Everything we do is together. We do a lot of reading, a lot of coloring, anything that can stimulate them*.

-Caregiver to child with prenatal drug exposure

*Positive outcomes*. Participants mentioned bonding, being happy, knowledge, soothing, laughing, healthy development, and connectedness as common positive outcomes to favorite home activities. Although positive outcomes were asked only about children, several participants shared experiencing positive outcomes, such as bonding, for themselves and their family.

*The interaction because some babies don’t get it and she gets it. She understands that we’re interacting with her so that comes really fast for her. A healthy family bond is a good outcome [of the interaction]*.

*Ever since he was little, my brother would sing his powwow music because he was a drummer and singer. So, he’d sing to him when he was little and he would instantly calm down and soothe him. Now, that’s him trying to sing to his little brother and cousin*.

- Biological parents to child with prenatal drug exposure

*Support, trust, understanding, love, communication, feeling like he’s part of everything, so being included*.

*How he can get used to our voices [when reading]. It’s their knowledge going and their brain going…He’ll sit there and have his eyes wide open, and he’ll smile on and off and he’ll get tired, too*.

- Biological parents to child without prenatal drug exposure

*She bonds through it. She laughs with the reading*.

*She’s learning how to share through playtime. She’s learning how to think of others’ feelings…she’s getting a sense of family that she really hasn’t had*.

-Caregiver to child without prenatal drug exposure

*Barriers*. Child being fussy, limited time, cost to purchase resources, and home-specific barriers were common barriers to engaging in home activities.

*My house is not big enough, my house is not clean enough. Time is a huge thing. Running out of books to read. A big barrier is temperature. So, if it’s too hot we do nothing. If it’s too cold, all we do is cuddle*.

*Having that family member that don’t like to be involved*.

- Biological parent to child with prenatal drug exposure

*Facilitators*. Income, community resources, having materials, and family or friend support were common supports to engage in home activities.

*With my mom or brother helping me with any activities for us to do is a big one. Or the materials like more books, games… Head Start or Early Childhood Services would help me get these materials that I need. I’m the only one that is supporting my kids. My mom helps when she can*.

- Biological parent to child without prenatal drug exposure

*I have older children as well so that is a support for younger ones to be able to do activities with them. Just having that family member present and maybe able to watch the little ones while we are able to do something with her age*.

-Caregiver to child with prenatal drug exposure

*Early Childhood, they always send out things that you can do with your kid…they also send out different kinds of snacks that you can make together. The WIC office does the same thing, like the food prep for babies, certain ages*.

- Biological parent to child with prenatal drug exposure

### Community bridge to traditional practices and identity

#### Familial barriers

Participants with and without a family history of substance use described a lack of familial exposure as an inherent barrier to knowing and practicing their cultural traditions and raised concerns of not being able to pass on such knowledge to their children. The interviewer (HR) was aware of such barriers in her tribal community and probed participants to share more when familial barriers were mentioned.

*It’s all about your last name. Like if your family is putting on a sweat, it’s usually only that certain family. My family has never really participated in any of that so it’s hard to find someone to help me get in, because you got to know someone to get help with sweats. I have never danced before and I didn’t even know where to start. Me and my daughter want to start dancing and I’m like, “I’ve never danced. I don’t even know if any of our families danced.” Kind of sucks. I don’t have anyone to reach out to who will sit down and teach me. My daughter is like, “Why don’t you just ask someone?” I’m like, “Who am I going to ask?” Because it’s kind of awkward if you don’t know anybody. Because it’s usually passed down through your family and like, “This is your family tradition, and this is the style that your grandma used to do.” So, when you don’t have that you don’t want to ask someone else to pass their family tradition to you unless you’re offered it from them, so it’s hard. I was telling my aunty, “Man, I wish we grew up dancing.” She’s like, “Yea, it sucks that our family grew up drinking and doing drugs.” It’s nice, because that’s not the thing anymore. The kids nowadays, like my age, want to restore traditions and get rid of the alcohol and the drugs. Now, that we’re trying to better this generation, it’s like, “Now, where do I go? How do I better this when all my family are drunks and drug addicts?”*

-Caregiver to a child without prenatal drug exposure

*Probably because she is a foster child. If she was in a biological family…I just think that when they’re foster children they are a little bit lost. They’re a part of our family, but they lost their family and some of that culture identity of just who they are goes away. I think they’re ignored. They know they’re not biologically my children, but they don’t know their biological parents always. I do think there is just a disconnect there*.

-Caregiver to a child with prenatal drug exposure

#### Community bridge

Although there were more biological parents to a child without prenatal drug exposure (n = 8) than biological parents to a child with prenatal drug exposure (n = 5), it was of interest that the latter participant group mentioned participating in more cultural activities (e.g., powwows, crafts and games, camps, native language program), and some outdoor and community activities (e.g., Farmer’s market, frequenting the park, berry-picking) compared to the former participant group. They also mentioned positive outcomes more often for cultural activities (e.g., cultural knowledge, connectedness) than both outdoor activities (e.g., knowledge, bonding) and home activities (e.g., soothing, knowledge, bonding). Although familial barriers to traditional practices and identity did occur, participants also mentioned community supports that act as a bridge to families having a sense of traditional identity and practice. The Salish language immersion program offered in early HeadStart is just one example that acts as a facilitator for cultural and traditional knowledge and practice.

*They went and dug bitterroots with HeadStart for the first time this year and that meant a lot to them, but that was the first time we’ve ever did that. I wish there were more things like that*.

*We go to the HeadStart Powwow. I know I can bring all of the kids. The teachers help me. They’ll go out and dance with the kids. They will hold the baby if I need them to. It’s actually the highlight of our year the HeadStart Powwow. They love it*.

-Caregiver to a child with prenatal drug exposure

*[Salish Language Nest]: He gets things that I’ve never got as a kid. So, he gets like introduction to the language, which will probably make it easier on him later in life to learn it*.

*Cultural knowledge, as well because they’ll [Home visitors] let me know of any events that will happen. I do feel comfortable talking with them about everything and coming up to my house. At first, I would meet them downtown, but after getting to know them, I let them come up to the house*.

-Biological parents to a child without prenatal drug exposure

## Discussion

Our study aimed to identify common positive family-child engagement activities particularly among children with prenatal drug exposure. Partnering with Early Childhood Services (ECS) and receiving guidance and support from the Community Advisory Team (CAT) members, elder/culture committees and the Tribal Council allowed us to apply community-driven, CBPR and strengths-based approaches that informed and shaped our overall study. The Tribal Nation houses several innovative programs that all participants had utilized, noting several positive outcomes for their children and themselves.

Early childhood intervention is critical to reduce symptom exacerbation and attenuate or prevent developmental dysfunction [28]. Interventions that incorporate early and continued family-child engagement promotes self-regulation, academics, reduced internalizing and externalizing behaviors and achieving developmental milestones [29–31]. All participants enjoyed attending and participating in local and nearby powwows/celebrations. They cited several positive outcomes for their child, like bonding, exercise, and connectedness to the culture and their family, which benefitted not only the child, but parents, caregivers, and family members. The frequently mentioned common, everyday family experiences (e.g., reading, speaking Native language, drumming) convey essential developmental supports that ultimately benefit the child. These everyday activities common across many Indigenous communities have been evidenced to support child resilience and adaptability when placed with a foster parent, and may also signal heightened emotional availability, an indicator of attachment security, between parents, family members and children [32, 33].

Participants shared on barriers and facilitators to activity participation. Feeling accepted at community and cultural events enabled participation whereas experiencing negative attitudes proved to be a hindrance to activity participation. Familial barriers to learning and participating in cultural and traditional practices were evident and participants indicated interest to learn their traditional practices. A potential solution not directly measured but related to familial barriers is community-level programs acting as a bridge to connect families and children to traditional ways of knowing and to their community. This community bridge along with feelings of acceptance at events may explain why families to children with prenatal drug exposure had the highest participation in cultural activities, for example. Participants with a family history of alcohol and drug use often mentioned family advocates and familial or friend presence or support were common facilitators to being able to learn and participate in their culture and community.

### Strengths and limitations

This study contributes to the literature on types of activities and factors that promote or inhibit family-child engagement, specifically for families that have children with prenatal drug exposure. The community partner, ECS, demonstrated exemplary capacity to engage in research. Family Advocates were well-connected with families and their support contributed to the recruitment and interview process completion within three weeks. The Tribal Nation’s investment helped to tailor our study tool by including questions that aligned well with the community context. Limitations included a small sample of caregivers with children that had prenatal drug exposure, and probable social desirability bias due to the potential for false reporting of drug use during pregnancy that, collectively greatly inhibit the generalizability of these findings.

## Conclusions

Native American communities are the nexus to introduce and sustain positive parenting practices that, in turn, support children’s physical, mental, emotional, social, and spiritual wellbeing. As demonstrated in our study, the tribal community set the foundation to support positive family-child engagement by promoting community cohesion. That is, several local events, programs, recreation opportunities, and services were made available to participants and their families. These community supports may be especially critical for families that had their traditional ways of knowing taken through colonization. Child bonding and cultural knowledge were heavily supported by community resources and benefited not only the child, but also the participants, family members, Elders, and the overall community. Family and friend presence, and local and free events and activities were primary drivers that encouraged participants and their families to frequent and participate in these family-friendly events and activities.

Future research using larger robust study designs is needed to measure Native American family and community practices as protective factors against maladaptive outcomes among children with prenatal exposure to drugs and/or alcohol. Implications from these findings are relevant to programs that serve families and children, especially those with a history of drug use. Being family-friendly, incorporating staff training that supports respectful interactions with clients, and acting as a “bridge” to foster community cohesion may have sweeping long-term benefits for families across generations.

## Supporting information

S1 File Caregiver interview guide. (PDF)
