# Supplementary material for: Identifying family-child activities among children with prenatal drug exposure in a Tribal Nation: Caregiver perspectives on barriers, facilitators and positive outcomes
Source: PLoS One. 2022 Sep 9;17(9):e0273989. doi: 10.1371/journal.pone.0273989 (PMC9462813; doi:10.1371/journal.pone.0273989)
Supplement: S1 File — (PDF) [file pone.0273989.s001.pdf]

Date, Time: ___________ Location: ___________ Audio or Hand-written (circle) PID: ____ Interviewer: ____

Table 1. Interview Guide: Domains and Subtopics for Family-Child Engagement Activities

---

Family type (circle one): Biol-parent drug-exp child Caregiver drug-exp child

Caregiver no drug-exp child Biol-parent no drug-exp child

DOMAIN: COMMUNITY/CULTURAL ACTIVITIES

PART I: AVAILABILITY OF COMMUNITY/CULTURAL ACTIVITIES

1.1 What are all of the community available to families that you can think of that are kid-friendly (0-3 years old)? (Probe: List programs, such as ECS, THD, Social Services, that host community events) (Prompt: basketball, football, fair).

1.2 What are all of the cultural practices that are available to your family that you can think of that are kid-friendly (0-3 years old)? (Prompt: round dances, powwow, sweats, language programs)

1.3 Of the cultural and community activities that you know about, which activities does your child get to do?

1.3.1 [If activities mentioned in 1.3] What positive outcomes, if any, does your child receive participating in [ACTIVITY]? (Prompt: connectedness, cultural knowledge)

1.4 Do you participate in any of the available services or program events through Early Childhood Services? (Probe: Services - Salish Language Nest, Early Headstart, Home Visiting-Education, Home Visiting-Family Advocate; Events: Cultural activities at parent meetings)

1.4.1 [If “Yes” to 1.4] What positive outcomes, if any, do you and your child receive participating in [ACTIVITY]? (Prompt: connectedness, cultural knowledge)

1.5 Do/Did you participate in any of the available services or programs through Bridge To Hope/Wrapped in Hope? (Probe: Bridge to Hope classes “Parent Curriculum”, counseling)

1.5.1 [If “Yes” to 1.5] What positive outcomes, if any, does/did you and your child receive participating in [ACTIVITY]? (Prompt: connectedness, knowledge)

PART II: BARRIERS TO COMMUNITY/CULTURAL ACTIVITIES

2.1 What barriers, if any, exist for you and your child to participate in these community or cultural activities? (Prompt: not know of engagement opportunities, not a part of family involved in cultural activities, time, cost, not from community) (Probe: Mention these events Baby Fair, W4W, M4W, Breastfeeding week)

2.2 Do you feel that your child receives enough supports from your community or cultural leaders? (Probe: Elders, community organizations)

2.2.1 [If “No” to 2.2] What are some reasons that you think your child does not get enough support? (Prompt: see 2.1 prompt)

2.3 What would be your top 3 community and/or cultural activities that you would like to do with your child?

PART III: FACILITATORS TO COMMUNITY/CULTURAL ACTIVITIES

3.1 What supports, if any, exist for your child and your or another family member to participate in community activities? (Prompt: feeling accepted, family member presence, transportation, childcare, family/friend(s), community resources, time and location, no-cost event)

3.2 What supports make it possible for your child to participate in cultural practices? (Prompt: See 3.1 prompt)

3.3 What supports, if any, are not available, but could support your child to participate in community or cultural activities? (Prompt: see 3.1 Prompt)

DOMAIN: OUTDOOR ACTIVITIES

PART IV: AVAILABILITY OF OUTDOOR ACTIVITIES

4.1 What are all of the common outdoor activities in your community that you can think of that are kid-friendly (0-3 years old)? (Prompt: Swimming, berry-picking, nature walks/hiking , backyard play, park, walk neighborhood, camping, fishing, community gardens, Farmer's market, fishing derby/Mack days)

4.2 What outdoor activities, if any, do you or another family member like to do with your child during the summer? (Prompts: See 4.1 prompt)

4.3 What do you think are the top 5 most important outdoor activities that you or another family member(s) do with your child? (Probe: Clarify on importance in terms of child well-being)

4.3.1 [If activities mentioned in 4.3] What do you think are the benefits, if any, that your child receives? (Prompt: knowledge, bond, happy, laugh, soothing)

PART V: BARRIERS TO OUTDOOR ACTIVITIES OF INTEREST

5.1 What barriers, if any, exist for you and your child to participate in these outdoor activities available in your community? (Prompt: safety, time, transportation, cost, attitudinal barriers, community resources, location)

5.2 What would be your top 5 outdoor activities that you and your child would like to do?

PART VI: FACILITATORS TO OUTDOOR ACTIVITIES OF INTEREST

6.1 What supports, if any, exist for your child and you or another family member to do outdoor activities? (Prompt: transportation, supportive neighbors, presence of family members, community resources)

6.2 What supports can you think of that are not available, but could support your child and you or another family member to do outdoor activities? (Prompt: see 6.1 Prompts, safe bike/walk paths)

DOMAIN: HOME ACTIVITIES

PART VII: AVAILABILITY OF HOME ACTIVITIES

7.1 What types of in-home activities do you or another family member(s) do with your child? (Prompt: Story-telling, read, sing, play, have dinner together)

7.1.1 [If activities mentioned in 7.1] What do you think are the benefits, if any, that your child receives? (Prompt: connectedness, cultural knowledge, happy, laugh, soothing)

7.2 What do you think are the top 5 most important at-home activities that you or another family member(s) do with your child? (Probe: Clarify on importance in terms of child well-being)

7.2.1 [If activities mentioned in 7.3] What do you think are the positive outcomes, if any, that your child receives? (Prompt: knowledge, bond, happy, laugh, soothing)

PART VIII: BARRIERS TO HOME ACTIVITIES

8.1 What barriers, if any, exist for your child and you or another family member to participate in at-home activities? (Prompt: time, cost, fussy, lack of family/friend member presence, overcrowded)

8.2 Do you feel that your child receives enough attention from you and family member(s)?

8.2.1 [If "No" to 8.2] What are some reasons that your child does not get enough attention? (Probe: Mention technology (phone, Netflix/TV, gaming) distraction, time, lack of family member presence, tired, out-of-house (gamble, shop))

8.3 What would be your top 5 inside activities that you and your child would like to do?

PART VIIII: FACILITATORS TO HOME ACTIVITIES

9.1 What supports make it possible for you or another family member and your child to do in-home activities? (Prompt: family member presence, supportive neighbors, community resources, personal/family income)

9.2 What other supports that you can think of that are not available, but could support you or another family and your child to do in-home activities? (Prompt: see 9.1 Prompts)

DOMAIN: FAMILY DEMOGRAPHICS

7.1 Please estimate how many family members spend time with your child in a typical week:

7.2 How many children (e.g., siblings, cousins) does your child have that live in the home?

7.3 What is your relationship to your child? (Prompt: biological father, foster parent, non-biological parent)

7.4 What is your child's primary race?

7.5 What is your primary race?

7.6 What is your age?

7.7 List all known drugs, if any, your child was prenatally exposed to:
